# Impedimetric Detection of Mutant p53 Biomarker-Driven Metastatic Breast Cancers under Hyposmotic Pressure

**DOI:** 10.1371/journal.pone.0099351

**Published:** 2014-06-17

**Authors:** Menglu Shi, Nataly Shtraizent, Alla Polotskaia, Jill Bargonetti, Hiroshi Matsui

**Affiliations:** 1 Department of Chemistry, Hunter College and the Graduate Center, City University of New York, New York, New York, United States of America; 2 Department of Biological Sciences Hunter College and the Graduate Center, City University of New York, New York, New York, United States of America; Georgia Regents University, United States of America

## Abstract

In cancer cells, the oncogenic mutant p53 (mtp53) protein is present at high levels and gain-of-function (GOF) activities with more expression of mtp53 proteins contribute to tumor growth and metastasis. Robust analytical approaches that probe the degree of metastasis of cancer cells in connection with the mtp53 activity will be extremely useful not only for establishing a better cancer prognosis but also understanding the fundamental mechanism of mtp53 oncogenic action. Here we assessed the influence of mtp53 in breast cancers to the mechanical property of breast cancer cells. Recently, ovarian and kidney cancer cell lines have been shown to have higher cellular elasticity as compared to normal cells assessed by monitoring the degree of deformation under hyposmotic pressure. To make fast detection in large scale, the impedance measurement was applied to monitor the swelling ratio of cells with time. The results showed that knockdown of mtp53 leads to decrease in cell swelling. In addition, by means of two types of impedimetric detection systems we consistently detected enhancement of impedance signal in mtp53-expressing breast cancer cells. Based on this observation we hypothesize that highly expressed mtp53 in metastatic mutant breast cancers can promote tumor progression by making cells more deformable and easier to spread out through extracellular matrix. The identification *via* the electric measurement can be accomplished within 10 minutes. All results in this report suggest that electric probing for the extent of the mtp53 expression of breast cancer cells may serve as a meaningful fingerprint for the cancer diagnostics, and this outcome will also have an important clinical implication for the development of mtp53-based targeting for tumor detection and treatment.

## Introduction

Constant efforts are being made to improve diagnostics and treatment of breast cancer, justified by the fact that certain subtypes of breast cancer do not respond to existing endocrine therapy. In this respect identification of novel biomarkers and their implication in diagnostics and targeted therapy remains a high priority. TP53 is mutated in 80% of basal-like breast tumors. Strong association between TP53 mutation status and aggressiveness of breast cancer is identified [1], and the mutant p53 (mtp53) up-regulates cholesterol biosynthesis and gives cells a more metastatic phenotype [2]. In normal cells, wild-type p53 is present at low levels due to fast turn over, and its stabilization is triggered by DNA damage followed by activation of signaling cascades that result in either DNA repair or apoptosis. The oncogenic mtp53 protein lacks this feature, is always stable, and facilitates genomic instability [3], [4]. Cancer cells express mtp53 proteins with a range of gain-of-function (GOF) activities, contributing to tumor growth and metastasis [5]–[8]. In agreement with this hypothesis, mice with mutant p53 developed a broad spectrum of tumors as compared to p53 knockout mice [9], [10]. Thus discovery of a novel approach that provides an assessment of metastatic potential of cancer cells in connection with the p53 activity, will be useful for not only establishing a more accurate cancer prognosis but also understanding the fundamental mechanism of mtp53 oncogenic action.

Recently, differences in mechanical and electrical properties between cancer cells and normal cells were identified by various analytical methods [11], [12]. One of the most robust approaches to screen them is to apply hyposmotic pressure and monitor the degree of cell deformation with impedance change on interdigitated electrodes (i.e., higher deformation leads to higher impedance signal). Cancer cells swell larger and faster due to their softer and elastic nature. Recently, ovarian and kidney cancer cell lines could be assayed with the increase of impedance signal on interdigitated electrodes even when cancer cells were spiked with the overwhelming number of normal cells in the samples [12].

In the present study we assessed the contribution of mtp53 in breast cancer cells to the mechanical property of breast cancer cells. We systematically depleted oncogenic mtp53 in breast cancer cells, hypothesizing that metastasized cells are more elastic and deform their cellular shape more dynamically than those cells whose mtp53 has been knocked down. The swelling event under hyposmotic pressure induced by adding water was indeed observed in aggressive mtp53-displaying breast cancer cells by fluorescence microscopy, and larger scale measurements of swelling cancer cells were accomplished by the impedance detection. The degree of elasticity of breast cancer cells can be correlated with the expression of mtp53, supporting the hypothesis that more metastatic cancer cells driven by mtp53 are softer and more deformable in hyposmotic pressure. All results in this report suggest that the mtp53 plays a pivotal role in increasing the flexibility of breast cancer cells and thus mtp53 expression coupled with impedance detection may serve as a meaningful fingerprint for the cancer diagnostics. These results will also have an important clinical implication for the development of mtp53-based targeting of detection and treatment.

## Materials and Methods

### Cell Culture

Cell lines (MDA-MB-231, MDA-MB-468, HCC70 and HCC1806) were purchased from American Type Culture Collection (ATCC) and grown in DMEM media, supplemented with 10% Fetal Bovine Serum and 1% penicillin-streptomycin, at 37°C humidified incubator at 5% CO_2_.

### Generation of Inducible mtp53 Knockdown Cell Lines

Constructs with shRNA for mtp53 or without the shRNA were generated and introduced into the MDA-MB-231 and MDA-MB-468 cells by retrovirus mediated gene transfer methods, as described elsewhere [2]. Briefly, STGM vector with miR30 based shRNA sequence and complimentary sequence to 2120–2139 of p53 (STGM-shp53) and rtTA plasmid were transfected into Phoenix packaging cells to generate retrovirus for infection of the target cells. The generated viruses were harvested and cells were co-infected with virus containing rtTA plasmid and the vectors. After selection with puromycin (STGM vector) and Hygromycin (rtTA), clonal cell lines were generated by limited dilution method. Doxocycline was added to induce the expression of the miR30-based shRNA, which could bind to the complementary fragment to p53 mRNA and initiate Dicer-mediated degradation of p53 mRNA. The resulting cell lines were named MDA-231.shp53 and MDA-468.shp53 respectively. Three clones are described in this work (MDA-231.shp53 1D10 and 2C9, and MDA-468.shp53 2F3). Two cell lines, MDA-MB-231 and MDA-MB-468, with empty vectors without p53-complementary oligonucleotide (STGM) but still containing miR30 based shRNA sequence were also generated. These two cell lines (MDA-MB-231 vector STGM and MDA-MB-468 vector STGM) and the parental MDA-MB-231 were used as negative controls. Cells were incubated with 8 µg/ml doxycycline (dox) for 6 days to knockdown mtp53.

### Western Blotting

Cells were lysed in RIPA buffer (0.1% SDS, 1% NP-40, 0.5% Deoxycholate, 150 mM NaCl, 1 mM EDTA, 0.5 mM EGTA, 50 mM Tris pH 8) containing 1 mM PMSF, 8.5 µg/ml Aprotinin and 2 µg/ml Leupeptin. SDS-PAGE was used to separate 50 µg of total protein cell extract per lane. Following electro-transfer to nitrocellulose membrane, immunoblotting was done with an anti-p53 antibody. Actin and lamin were used as loading controls.

### Monitoring Cell Swelling Using Inverted Epifluorescence Microscopy

Cells were seeded in complete medium on BD poly-D-lysine multiwell plates and cultured for 1 h. Wheat germ agglutinin conjugate (0.5 mg/mL, Invitrogen) was added for 20 min to label plasma membrane of the cells. Subsequently, the medium was replaced by deionized water to induce the hyposmotic stress. Cells remained attached to the plate surface during the swelling experiment. The increase and the variability of the diameter were determined by a series of Fluorescence micrographs taken by a Nikon Eclips TE200 inverted epifluorescence microscope. The mean increase in volume during swelling was analyzed using ImageJ software for 40 cells before and after undergoing the hyposmotic stress.

### Impedimetric Measurements for the Detection of Cell Deformation

The single-shell theory suggests that since cells are surrounded by dielectric shells in low permittivity with conductive electrolyte cores they can be treated as insulated spheres [13]. When an alternating current (AC) field with the frequency less than 1 MHz is applied on electrodes, the deposition of such insulated cells alters the distribution of electric-field lines on interdigitated electrodes, leading to the impedance change around the electrodes [14]. When the volume of insulating objects becomes larger on electrodes, the deviation of the electric-field lines increases and then this structural change sensitively influence the electric environment around the electrode [15], leading to the enhancement of impedance signal. To measure cell deformability using interdigitated electrodes, 218 polysilicon fingers (1600 µm-long, 3 µm-wide and a 3 µm-spacing with each other) were fabrication as described elsewhere [15]. The electrodes were incubated with polylysine (0.1 mg/mL, poly-D-lysine hydrobromide) for 30 min, rinsed with deionized water and dried under nitrogen gas to ensure adhesion of cells on the transducer surface. Cancer cells were seeded on top of electrode in a 5 µl volume and incubated for 30 min in a humidified chamber. Following the incubation, the electrode was rinsed three times with deionized water and real-time impedimetric measurements by the polysilicon transducer were recorded at 20 kHz, 10 mV.

For the analysis of deformability using a commercial impedance analyzer, xCELLigence, a 16-well plate (e-plate 16, ACEA Biosciences, Inc.) was incubated with 100 µl of poly-L-Lysine for 30 min, rinsed with deionized water and dried in a cell culture hood under UV light. On a poly-L-Lysine coated plate in quadruplicate, 2000 cells were plated per well. Following 2 hours of cell stabilization on plates at 37°C, culture media was replaced with dH_2_O and the impedance measurement was recorded every 2 seconds for 10 minutes. Impedance values were automatically converted to Cell Index (CI), relative impedance change at every measurement point. The xCELLigence system measures impedance at three discrete frequencies, 10 kHz, 25 kHz, and 50 kHz and it plots averaged impedance CI values among these frequencies.

## Results and Discussion

**Figure 1:**
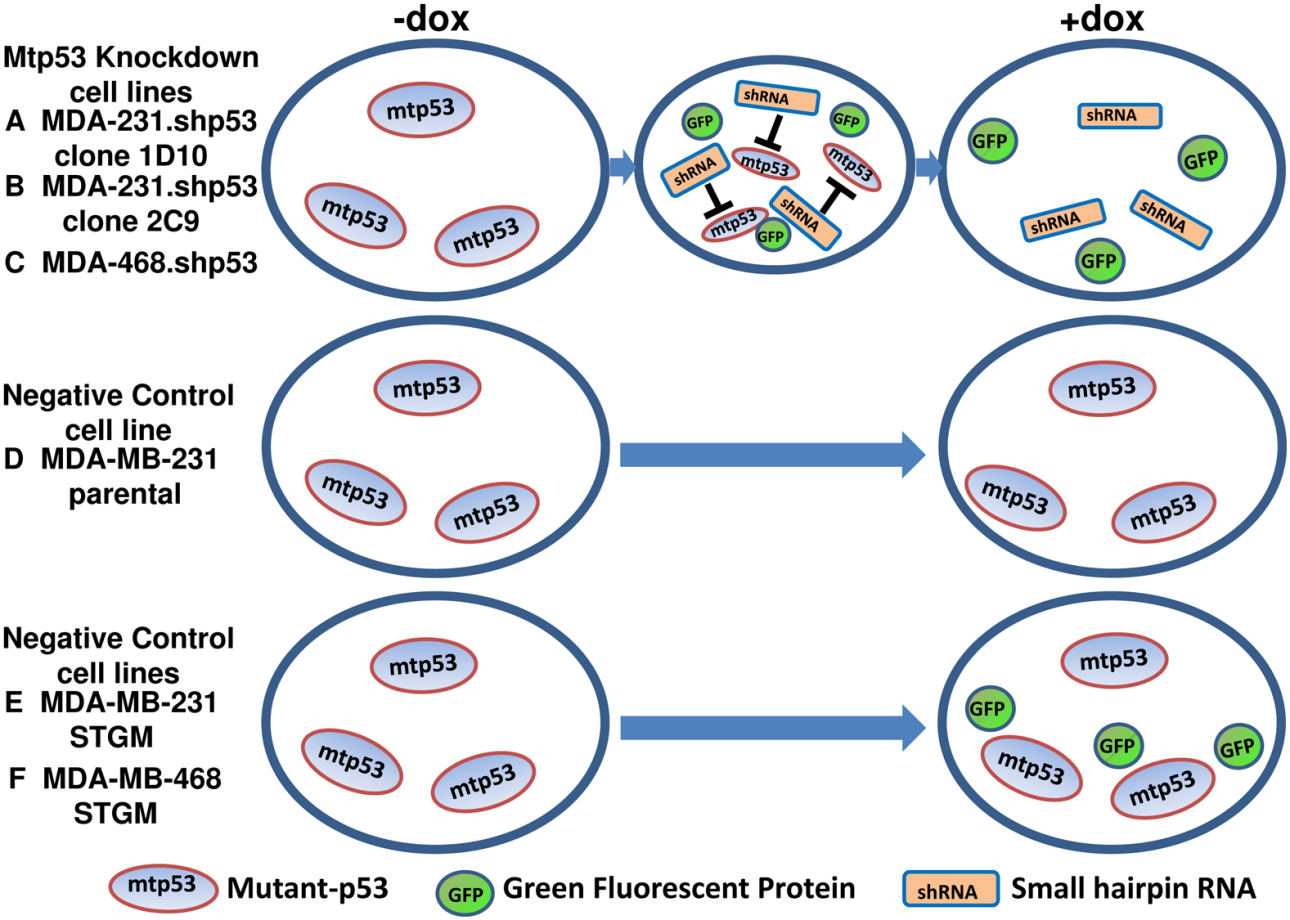
Schematic illustration of the response of various breast cancer cell lines to doxycycline treatment. The degree of the expression of mtp53 in shp53 cell lines (Figures 1 A–C) can be controlled with the treatment of dox, while the mtp53 expression in parental and vector-STGM cell lines remains unchanged.

The aim of our work is to probe changes in the mechanoelastic property of breast cancer cells associated with the expression of mtp53. We hypothesized that high expression of mtp53 in two types of breast cancer cells (MDA-MB-468 (with mtp53 R280H) and MDA-MB-231 (with mtp53 R273L)) leads to softening of cell structure and the hyposmotic pressure induces swelling of these cells in contrast with ones devoid of mtp53. Thus we expected that the removal of mtp53 would decrease elasticity and revert cells to a stiffer normal phenotype. To test this hypothesis, we induced depletion of mtp53 in shRNA-mediated knockdown cell lines, MDA-231.shp53 (Figure 1-A, B) and MDA-468.shp53 (Figure 1-C), and analyzed their mechanoelastic change before and after the knockdown. The shRNA cancer cells were generated through miR30-based knockdown of mtp53 induced by doxycycline. While doxycycline controls the degree of the expression of mtp53 in shp53 cell lines (Figures 1-A–C) it does not influence the mtp53 expression in parental and vector-STGM cell lines (Figures 1-D–F); The engineered MDA-MB-231 vector STGM cells and MDA-MB-468 vector STGM cells express the same proteins as MDA-MB-231 and MDA-MB-468 except without mtp53.shRNA and thus doxycycline cannot knockdown mtp53. Therefore, the parental MDA-MB-231, MDA-MB-468 vector STGM and MDA-MB-231 vector STGM cells served as negative controls. And the cellular mechanical property should also reflect this metastatic behavior change based on our hypothesis.

**Figure 2:**
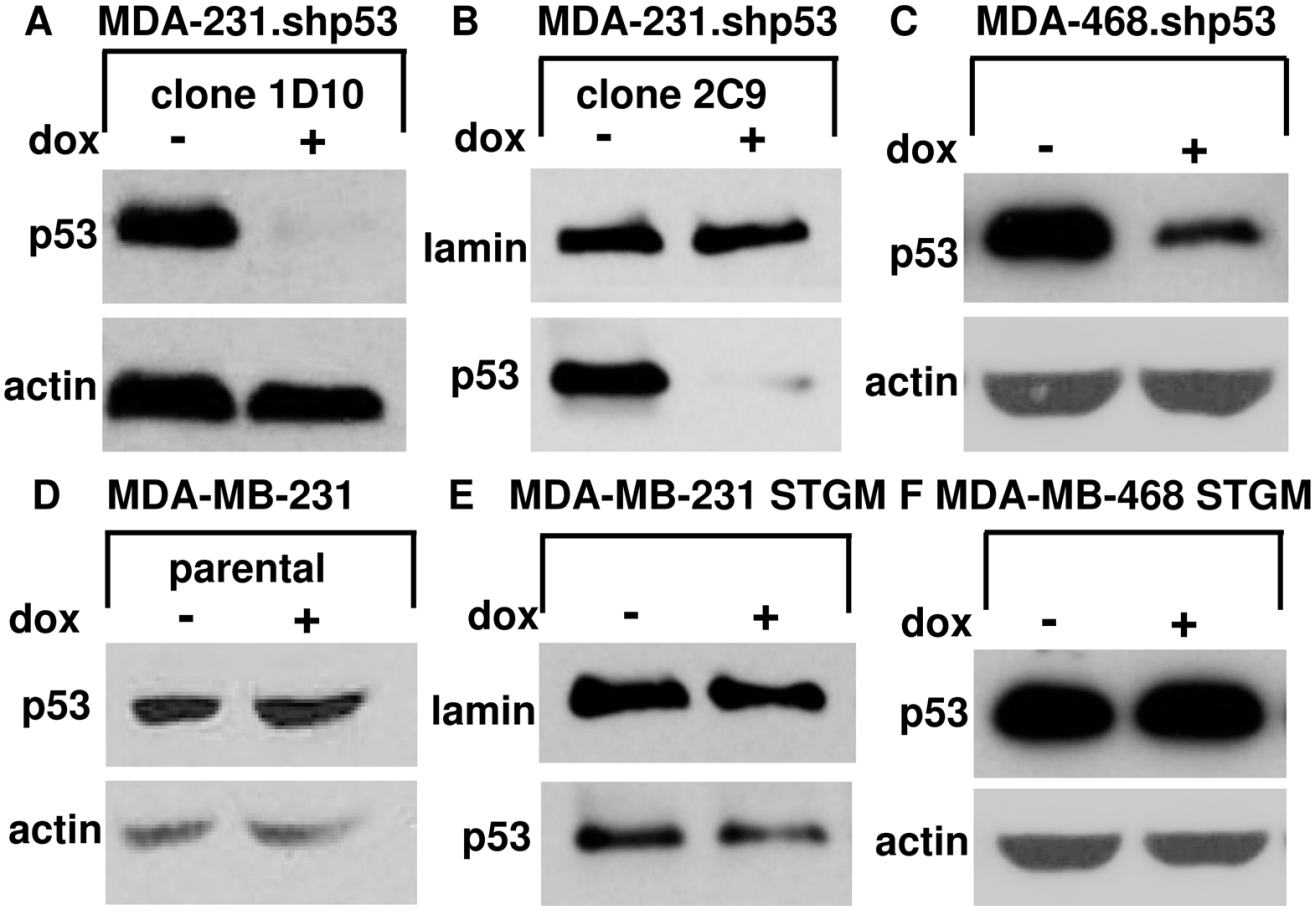
Western blot analysis of the expression of mtp53 in cells with or without the treatment of dox. A. MDA-231.shp53 (clone 1D10), B. MDA-231.shp53 (clone 2C9), C. MDA-468.shp53 (clone 1F5), D. MDA-MB-231 parental, E. MDA-MB-231 STGM, and F. MDA-MB-468 STGM breast cancer cells. Actin is control for A, C, D, F and lamin is control for B and E.

**Figure 3:**
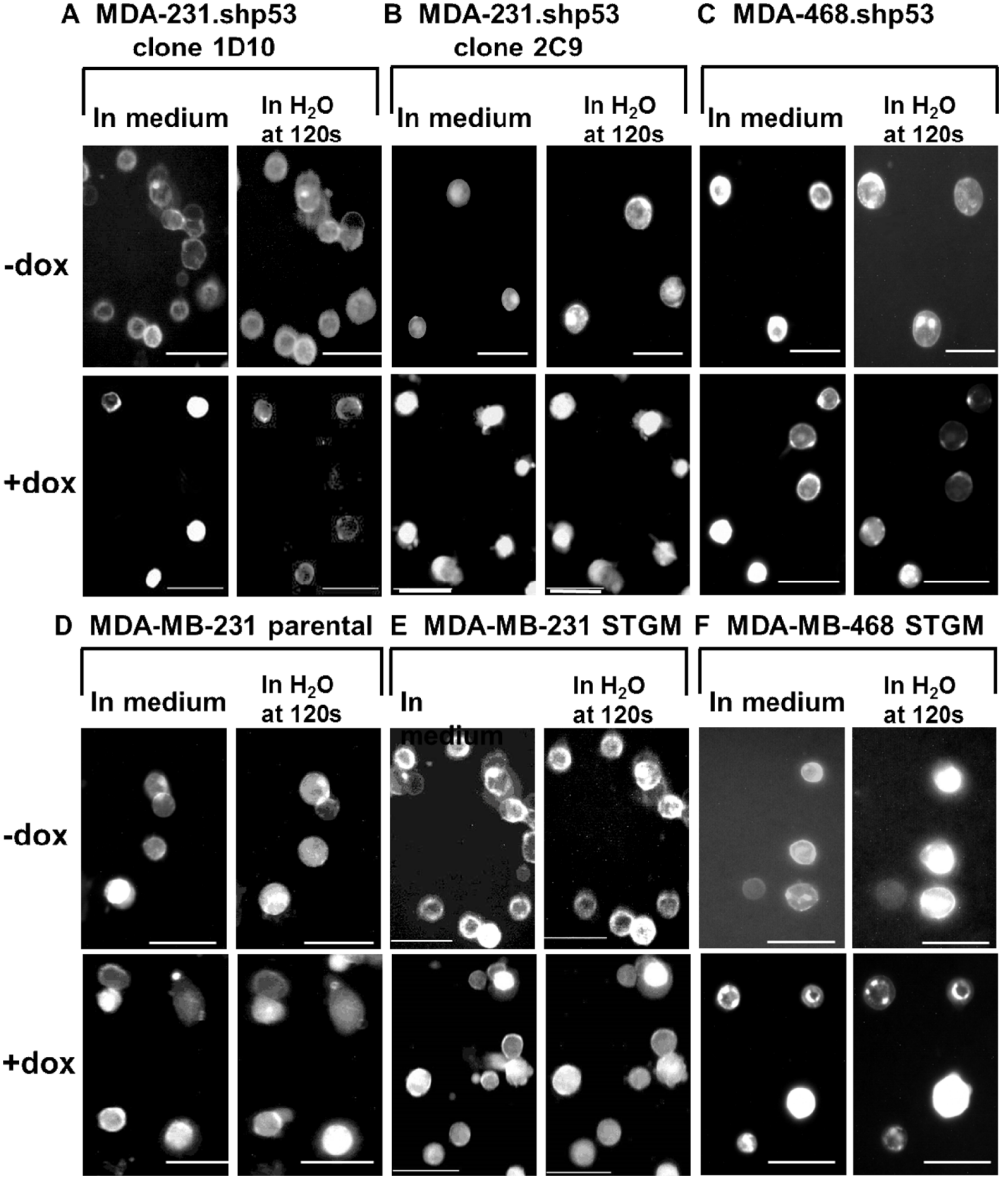
Fluorescence micrographs of stained plasma membranes of various cells. Cells were examined in the culture medium and then in deionized water at 120-231.shp53 (clone 1D10), B. MDA-231.shp53 (clone 2C9), C. MDA-468.shp53 (clone 2F3), D. MDA-MB-231 parental, E. MDA-MB-231 STGM, and F. MDA-MB-468 STGM breast cancer cells. Scale bar = 60 µm.

First, the expression of mtp53 in each cell line with or without the treatment of doxycycline was examined by western blot analysis. In the MDA-231.shp53 and the MDA-468.shp53 (Figures 2-A, B, and C), harboring the shRNA designed to knockdown mtp53, the levels of mtp53 protein was decreased after the addition of doxycycline. Figures 2-D, E, and F show the expression of mtp53 in MDA-MB-231 parental, MDA-MB-231 STGM and MDA-MB-468 STGM cells remained at the same level before and after the doxycycline addition. Then, we analyzed change in volume of each individual cell *via* application of hyposmotic pressure with or without the treatment of doxycycline by fluorescence microscopy. After plasma membranes of these cells were stained by a fluorescent dye, wheat germ agglutinin conjugate, and immersed in deionized water for the hyposomosis, fluorescence micrographs showed that MDA-231.shp53 and MDA-468.shp53 cells increase diameters significantly by 35±5%, 37±4% and 38±6%, respectively, after adding deionized water for 120s (Figures 3-A, B and C). When these cells were treated with doxycycline for the reduction of mtp53, the diameter change of MDA-231.shp53 and MDA-MB-468.shp53 cells was significantly reduced as compared to the non-doxycycline-treated cancer cells to 22±4%, 21±4%, 13±3%, respectively. These trends suggest that higher expression of mtp53 on the breast cancer cell lines has correlation with larger volume changes as the hyposmotic pressure was applied. As a control, we confirmed that there is no difference in the swelling ratio of MDA-MB-231 parental in the presence of doxycycline after adding water (37±6% with no doxycycline, 37±7% with doxycycline, Figure 3-D), consistent with the hypothesis that the cause of elastic nature is due to the expression of mtp53 rather than the doxycycline treatment. Both MDA-MB-231 STGM and MDA-MB-468 STGM cells swell in the same expansion percentage before and after adding doxycycline (Figures 3-E and F), further supporting that the reduced elasticity of shp53 cell lines is specifically caused by mtp53 knockdown, not by the presence of mir-30 sequence and STGM vector. Recently, the higher degree of metastasis can be correlated with the higher mtp53 expression in breast cancer cells [8], and thus cancer cells that have higher metastatic potential due to expression of mtp53 could have altered mechanoelastic properties and be more aggressive due to increased elasticity.

**Figure 4:**
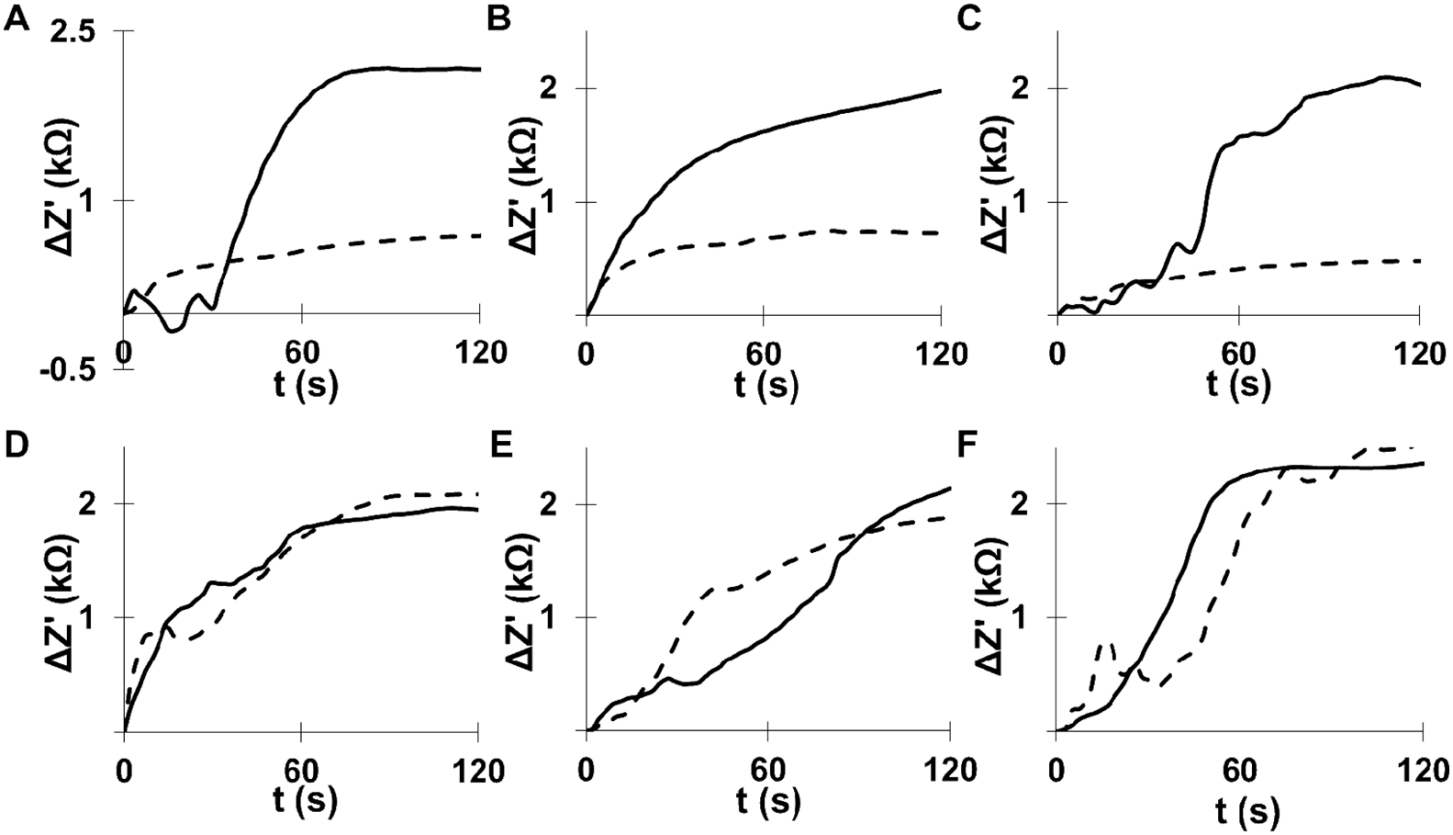
Variations of impedance (Z’) as the function of time resulted from the swelling under hyposmotic stress. With regard to all the samples, 1000 cancer cells with dox (solid lines) or without dox (dotted lines) were incubated with electrodes for 30 min. A. MDA-231.shp53 (clone 1D10), B. MDA-231.shp53 (clone 2C9), C. MDA-468.shp53 (clone 2F3), D. MDA-MB-231 parental, E. MDA-MB-231 STGM, and F. MDA-MB-468 STGM breast cancer cells.

To confirm this trend of structural transformation with a larger number of cells, we investigated the impedance variation of mtp53-containing breast cancer cells on the order of 1,000 cells before and after applying hyposmotic pressure. Previously, ovarian and kidney cancer cell lines were confirmed to increase impedance values as these cells were swelled by mixing with water [12] and here we used the same protocol to correlate the elastic structural characteristic with the degree of mtp53 expression and the metastatic feature. After interdigitated electrodes were coated with polylysine for cell adhesion, 5 µL of cell suspension was incubated on top of the electrode and then deionized water was added to trigger the swelling *via* the increase of hyposmotic stress. Impedance changes in MDA-231.shp53 and MDA-468.shp53 cells were measured over time at 20 kHz, the same protocol previously optimized for the sensitive detection of cancer cells [12]. The real part of the impedance (Z’) increased rapidly for all of these cells, indicating that these cells are elastic. However, after addition of doxycycline their impedance values decreased and the difference became clear after 60 s of swelling time due to the reduced elasticity (Figures 4 -A, B, and C). All control cell lines, MDA-MB-231 parental, MDA-MB-231 STGM and MDA-MB-468 STGM, also showed the same impedance values through the swelling time with and without doxycycline (Figures 4-D, E, and F), meaning that these cells swelled in the same volume expansion % regardless of the presence of doxycycline. The detection limit of breast cancer cells with and without mtp53 expression is 2 cells/µL with two times the standard deviation of blank signal in the swelling time of 120 s. Thus, this comparison suggests that breast cancer cells with different levels of mtp53 could be screened by the impedance variation induced by the volume change under the hyposmotic pressure in the range of swelling time between 60 s and 120 s where the impedance difference between mutant breast cancer cells and the one with mtp53 knockdown is significant. It should be noted that the size of MDA-231.shp53 increased with the incubation time and reached the plateau at 60 s of the incubation time (Figure S1), agreeing with the impedance change profile in Figure 4. From this observation, the cell expansion limit in an average size change % was determined as 38%. The agreement between the size change and the impedance profiles indicates that there is no major loss of cancer cells *via* lysis in the range of impedance measurement times; because no significant size change is observed after 60 s of incubation time under hyposmotic pressure, the impedance values of cells should be decreased if many cells are lost by lysis. However, the decrease of impedance values is not observed as shown in Figure 4, supporting that lysis does not interfere with the impedance measurement. In addition, a major loss of cells is not recognized in a series of fluorescence images under osmosis. The feature for no size change of cells after a few minutes of the osmosis is consistent with cells returning to iso-osmotic medium, which was also previously observed in other cells and mutants [16].

**Figure 5:**
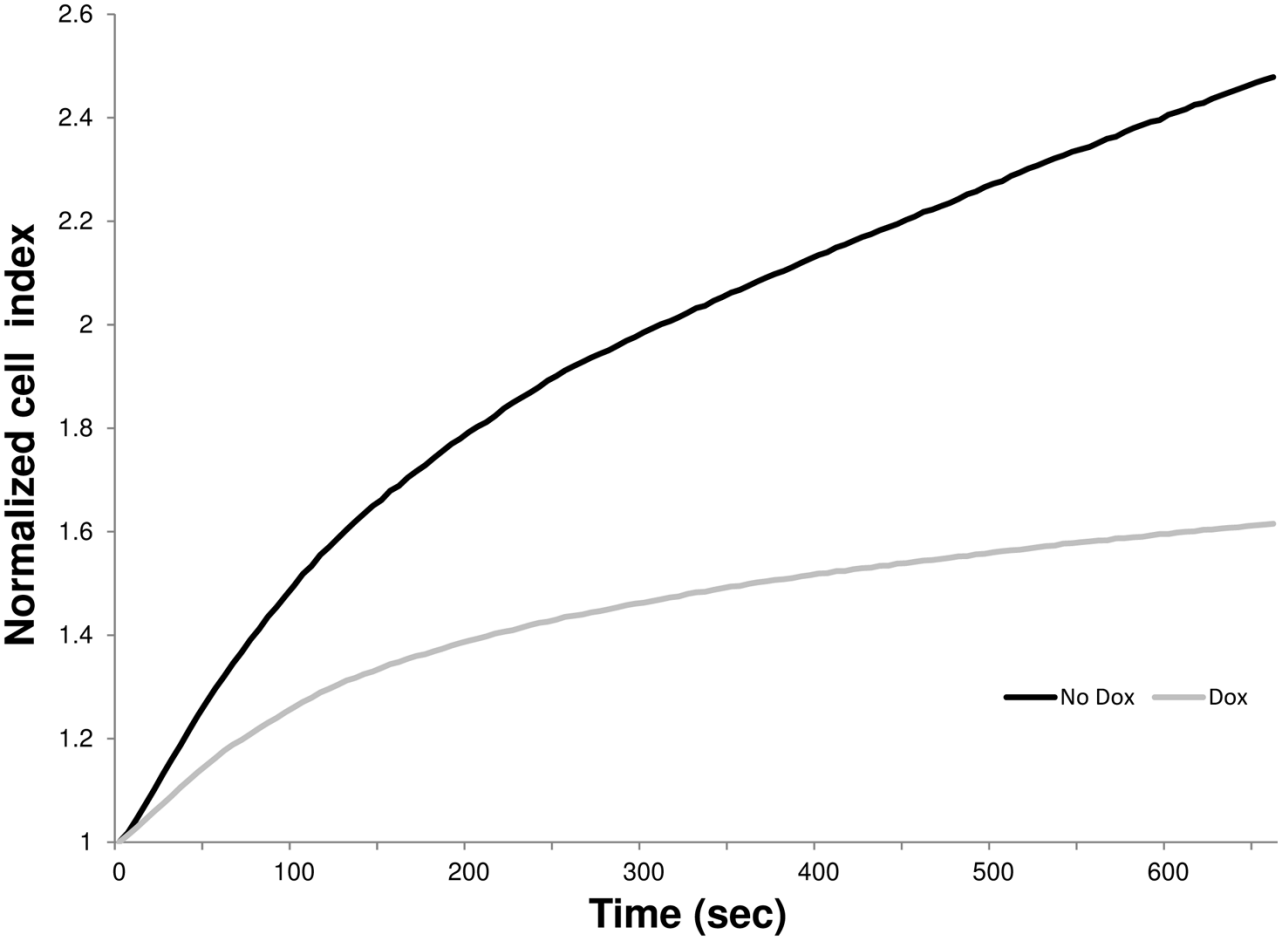
Average variations of the impedance (CI) as the function of time resulted from the swelling. MDA-468.shp53 cell line (black) and the one treated with doxycycline (gray) were measured under hyposmotic stress in three sets of 16-well plates simultaneously using xCelligence system.

In order to further confirm the trend we observed between the expression of mtp53 and the elasticity of breast cancer cell lines with larger sample sets, impedance changes of these cells were analyzed by a commercially available impedance analyzer, xCELLigence impedance analysis system (ACEA Biosciences, Inc.), containing three sets of 16-well plates for simultaneous measurements. With the same protocol using a short cell attachment phase followed by the addition of water, Cell Index (CI) of swelling was measured every 2 seconds over a period of 10 minutes. The electric swelling assay, using xCELLigence system was performed in a CO_2_ incubator, the standard detection compartment of xCELLigence reducing the impedance fluctuation. Consistently with our initial results a significant increase of the impedance change was detected in MDA-468.shp53 cells expressing mtp53 as compared to the cells where mtp53 was knocked down (Figure 5). This result confirms that mtp53 knockdown in the MDA-468.shp53 cell line partially restored cellular rigidity with higher statistical confidence.

**Figure 6:**
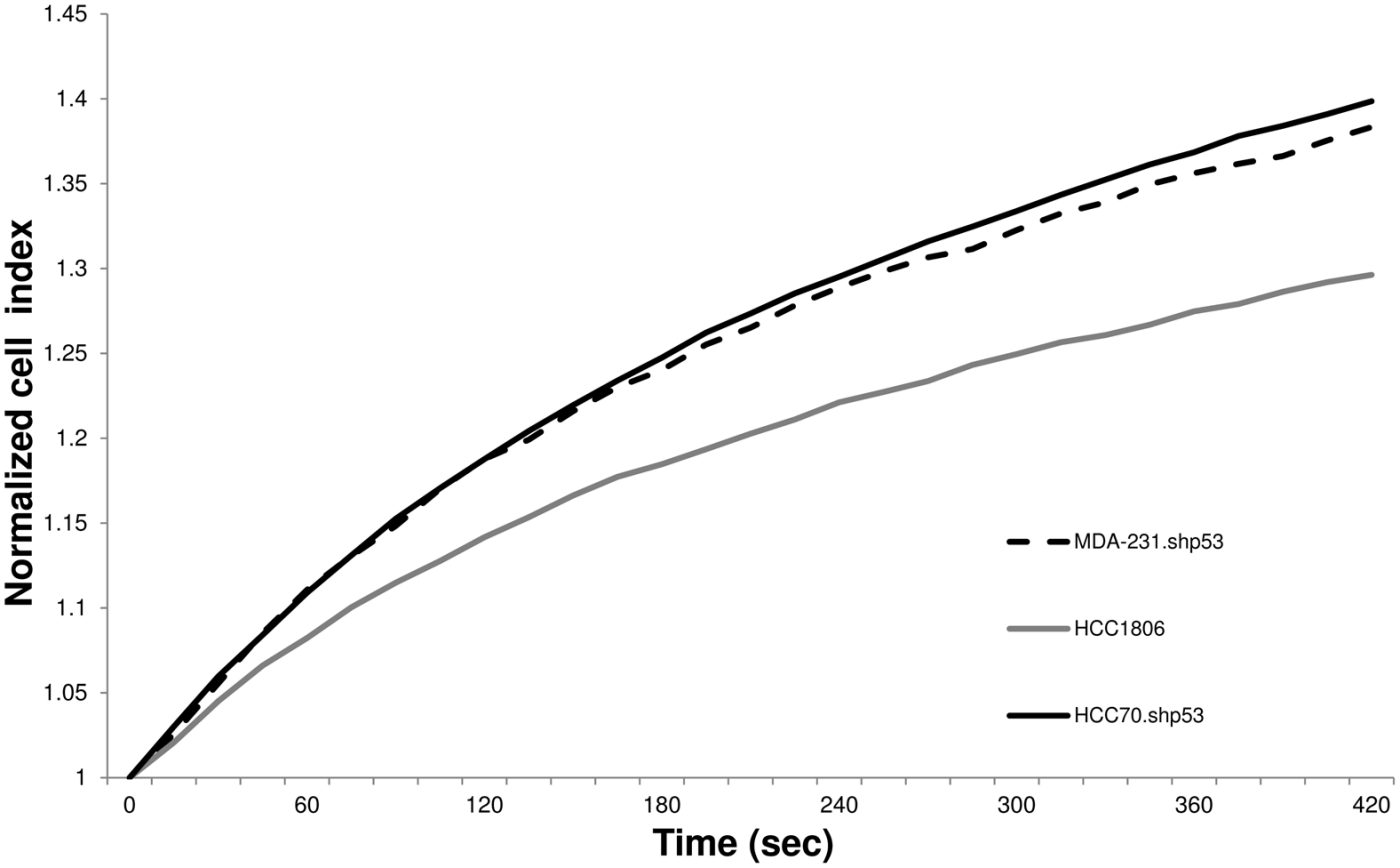
Average variations of the impedance (CI) as the function of time resulted from the swelling. Mtp53-expressing cell lines (MDA-231.shp53 (clone 1D10) (dotted black), HCC70 (solid black)) and no p53-expressing cell line (HCC1806, solid grey) were measured under hyposmotic stress using xCELLigence system.

To further correlate the origin of impedance signal enhancement with mtp53 expression, we performed impedance studies on two additional breast cancer cell lines, the breast cancer cell line HCC70 expressing mtp53 and the breast cancer cell line HCC1806 that does not express any p53 protein. We found that HCC70 cells generated a substantially higher impedance signal during cell swelling as compared to HCC1806 cells without p53 expression using both detection methods (Figure 6). Because the expression of mtp53 is independent of the effect of the shRNA and STGM vector for these cells, this result supports that the degree of mtp53 expression is related to the impedance signal in breast cancer cells and this correlation is valid with and without the triggering process of shRNA expression.

In summary, these studies provide new insights into understanding the role of mtp53 in the increased elasticity of cell structure in cancer cells. The cell swelling ratio under hyposmotic pressure is proportional to the impedance signal enhancement on interdigitated electrodes. This approach could be applied to characterize when breast cancer cells with high mtp53 expression (MDA-231.shp53 and MDA-468.shp53) and to observe reversion to more normal and stiffer isotypes as mtp53 is knocked down by shRNA. Moreover, our work revealed that other breast cancer cell lines HCC70 and HCC1806, which originally have different expression levels of mtp53, could be distinguished by their impedance signal. This outcome demonstrates that the expression of mtp53 positively correlates with the deformability of cancer cells, which can potentially be applied as an inherent cell marker for metastatic competence [17]. The described impedance-based sensor can be used to test effect of mtp53-targeted drugs with respect to the change in ability of cancer cells to exhibit tumorigenic deformability.

## Supporting Information

Figure S1 **Percent variation of the diameter of MDA-231.shp53 (clone 1D10) breast cancer cells in deionized water.** The swelling % of the breast cancer cells was determined by fluorescence microscopy.
(TIF)
