# Supplementary figures and images for: Impedimetric Detection of Mutant p53 Biomarker-Driven Metastatic Breast Cancers under Hyposmotic Pressure
Source: PLoS One. 2014 Jun 17;9(6):e99351. doi: 10.1371/journal.pone.0099351 (PMC4060997; doi:10.1371/journal.pone.0099351)

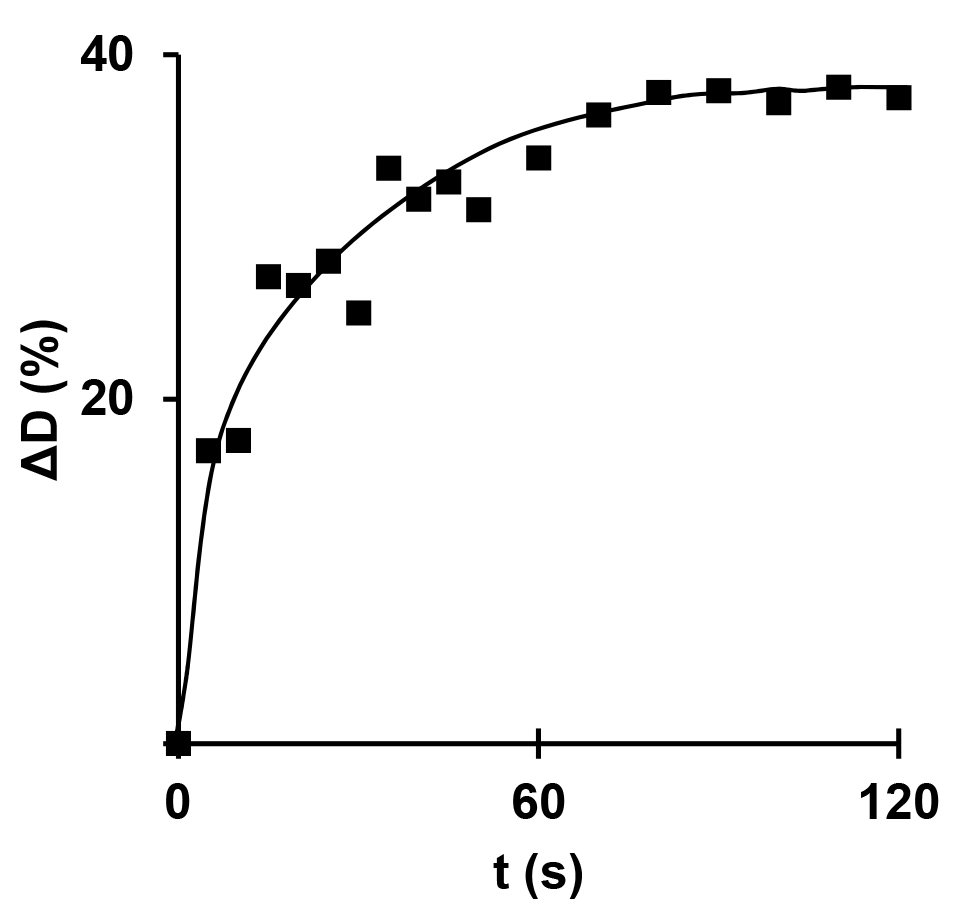

Supplement: Figure S1 — Percent variation of the diameter of MDA-231.shp53 (clone 1D10) breast cancer cells in deionized water. The swelling % of the breast cancer cells was determined by fluorescence microscopy. (TIF) [file pone.0099351.s001.tif]
